# The cost-effectiveness of unilateral cochlear implants in UK adults

**DOI:** 10.1007/s10198-021-01393-y

**Published:** 2021-11-02

**Authors:** Henry Cutler, Mutsa Gumbie, Emma Olin, Bonny Parkinson, Ross Bowman, Hafsa Quadri, Timothy Mann

**Affiliations:** 1 Macquarie University Centre for the Health Economy, Sydney, Australia; 2 Health Technology Analysts, Sydney, Australia; 3 Cochlear Limited, Sydney, Australia

**Keywords:** Cost–utility, Cochlear implant, Hearing aid, Hearing loss, Economic evaluation, D61, I18, I19

## Abstract

**Objective:**

The National Institute for Health and Care Excellence (NICE) updated its eligibility criteria for unilateral cochlear implants (UCIs) in 2019. NICE claimed this would not impact the cost-effectiveness results used within its 2009 technology appraisal guidance. This claim is uncertain given changed clinical practice and increased healthcare unit costs. Our objective was to estimate the cost-effectiveness estimates of UCIs in UK adults with severe to profound hearing loss within the contemporary NHS environment.

**Methods:**

A cost–utility analysis employing a Markov model was undertaken to compare UCIs with hearing aids or no hearing aids for people with severe to profound hearing loss. A clinical pathway was developed to estimate resource use. Health-related quality of life, potential adverse events, device upgrades and device failure were captured. Unit costs were derived mostly from the NHS data. Probabilistic sensitivity analysis further assessed the effect of uncertain model inputs.

**Results:**

A UCI is likely to be deemed cost-effective when compared to a hearing aid (£11,946/QALY) or no hearing aid (£10,499/QALY). A UCI has an 93.0% and 98.7% likelihood of being cost-effective within the UK adult population when compared to a hearing aid or no hearing aid, respectively. ICERs were mostly sensitive to the proportion of people eligible for cochlear implant, discount rate, surgery and device costs and processor upgrade cost.

**Conclusion:**

UCIs remain cost-effective despite changes to clinical practice and increased healthcare unit costs. Updating the NICE criteria to provide better access UCIs is projected to increase annual implants in adults and children by 70% and expenditure by £28.6 million within three years. This increased access to UCIs will further improve quality of life of recipients and overall social welfare.

## Introduction

Around 11 million people in the UK live with permanent hearing loss, most of which results from age related damage to the cochlear due to environment and genetic factors [1]. A unilateral cochlear implant (UCI) can improve hearing in people with severe to profound sensorineural hearing loss. Speech recognition is better with cochlear implants compared to hearing aids for adults with post-lingual severe to profound bilateral hearing loss, regardless of age [2–8]. While pre-lingual deafened people derive significant benefits from cochlear implants, people with post-lingual severe to profound hearing loss receive the greatest improvements in speech perception [8, 9]. Gains in speech perception scores are greatest in the first few months, but performance continues to improve over time [10, 11].

Improved hearing from using a UCI also improves quality of life. People have reported improvements in the Geriatric Depression Scale [12, 13], improved mental health and social functioning in the Short Form Health Survey (SF-36) [10, 14–16], improved health-related quality of life as measured by the Health Utilities Index Mark 3 (HUI3) [11], and improved general wellbeing, as measured by the Glasgow Benefit Inventory [17, 18].

Many people in the UK with severe to profound sensorineural hearing loss have missed out on receiving a UCI despite their potential benefits. One study concluded five% of adults eligible for a cochlear implant had received one within the UK, arguing that low provision and utilisation resulted from limited audiological awareness and screening programs [19]. Another study argued that NICE guidance was too restrictive compared to other countries [20]. Research also suggested that the Bamford–Kowal–Bench (BKB) sentence test was not appropriate for assessing the potential benefits from a cochlear implant for some users, particularly pre-lingual deaf adults, people without English as their native language, and those with low-frequency residual hearing [21].

Previous National Institute for Health and Care Excellence (NICE) guidance stated that an adult must have severe to profound hearing loss (threshold greater than 90 dB HL at 2 or more frequencies (500, 1000, 2000 and 4000 Hz) and not receive adequate benefit from acoustic hearing aids for a cochlear implant to be funded by the National Health Service (NHS). NICE defined adequate benefit from acoustic hearing aids in adults as a score of 50% or greater using BKB sentence testing at a sound intensity of 70 dB SPL [22].

NICE updated its guidance in March 2019 [23]. Severe to profound hearing loss became defined using a threshold of equal to or greater than 80 dB HL at 2 or more frequencies (500, 1000, 2000 and 4000 Hz) bilaterally without acoustic hearing aids. Adequate benefit from acoustic hearing aids also became defined for adults as a phoneme score of 50% or greater on the Arthur Boothroyd (AB) word test presented at 70 dBA.

Updated eligibility criteria are projected to increase annual access to UCIs by around 70% and cost around £28.6 million annually within three years [24]. While studies that have evaluated the cost-effectiveness of cochlear implants in the UK have generally found UCIs are cost-effective [5, 30–33], the cost-effectiveness of UCIs has not been explored since 2009, when a NICE Health Technology Assessment Report was produced [25], and relied upon to develop the original guidance. That study primarily relied on estimated costs that could be considered an unreliable representation of costs within clinical settings today. Costs were estimated from a study of five hospitals in the NHS between 1992 and99, which apportioned administration costs across planned patient contact hours sourced from clinicians to estimate resource unit costs per hour of contact, and then projected costs using a declining exponential function [5]. It also used costs derived from consultation with clinicians and hospital accountants for imaging, surgical sessions, radiography, cochlear implant system and repairs, and a hospital stay [5] These costs were converted from euros to pounds and inflated to 2005–2006 prices [25].

The cost-effectiveness of UCIs in the UK adult population has not been investigated since. NICE noted that changes to the eligibility criteria within its 2019 technology appraisal guidance update would not impact the cost-effectiveness results presented within the NICE Health Technology Assessment Report. It reasoned that the original cost-effectiveness estimates included the additional population defined under the new eligibility criteria and that device costs had since declined [26].

This claim is uncertain given changes in the NHS environment since the NICE Health Technology Assessment Report was completed. While device costs have recently decreased, they represent only around 39% of the total cost associated with a UCI [25], with the remaining proportion associated with assessment, surgical implantation, activation and tuning, rehabilitation and maintenance. Continual increases in the Hospital and Community Health Services (HCHS) index suggest unit costs within the NHS have increased by 37% between 2005–06 and 2019–2020 [27, 28]. Increased unit costs may have increased the incremental cost-effectiveness ratio (ICER) beyond the NICE cost-effectiveness threshold. If this has occurred, extending access to UCIs will improve health outcomes for recipients, but will reduce social welfare given the additional resource could be spent elsewhere in the NHS with potentially greater returns [29].

Our study aims to evaluate the cost-effectiveness of UCIs compared to hearing aids and no hearing aids in UK adults with severe to profound hearing loss within a contemporary NHS environment. Our target population includes adults who had previously received benefits from a hearing aid and adults who had not. Our study represents the first alternative cost-effectiveness estimate of UCIs compared to that presented within the NICE Health Technology Assessment Report in 2009. It is timely given new guidance is expected to significantly increase access to UCIs for UK adults with severe to profound hearing loss.

We update previous results from the NICE Health Technology Assessment Report in three important ways. First, we use a contemporary UCI clinical pathway for the UK developed in consultation with clinicians. Our pathway therefore inherently captures change in UCI clinical pathways over the last two decades. While we do not quantify this change directly, there is evidence that the clinical pathway has changed that could significantly impact costs. For example, costs within the NICE Health Technology Assessment Report relied on clinical estimates of time spent in hospital after cochlear implant surgery, which was estimated to be 72 h [5]. Today most adults receive a UCI in an outpatient setting within the UK, or spend one night in hospital for observation.

Second, our study differs from the NICE Health Technology Assessment Report by relying on current NHS reported costs and tariffs derived from the NHS National Schedule of Reference Costs [30]. This presents national average costs across all NHS providers, and therefore capture all hospitals providing UCIs. It also accounts for changes in the price of UCIs, which are expected to have occurred considering UCI devices have been miniaturised and undergone significant technological change to processors and electrodes since the mid 1990s [31]. Third, our study accounts for changes in short- and long-term adverse events associated with UCIs that has occurred through improvements in surgery process and improved cochlear implant devices. We rely on more recent clinical studies that have evaluated adverse events from cochlear implant surgery and UCIs.

## Methods

A UCI is expected to improve hearing ability over the remaining life of a recipient. There are also ongoing lifetime costs, such as sound processor upgrades, consumables (cables and batteries), audiologist consultations for maintenance and upgrades, and costs to explant and reimplant a cochlear implant if the device fails or due to medical reasons.

A Markov model was employed to capture the chronic nature of severe to profound hearing loss in adults, treatment pathways and health outcomes associated with UCIs and hearing aids. It allowed for death from other causes (which increases in likelihood as the cohort ages) to be incorporated.

Parameter estimates were incorporated as mean values with an associated prior distribution representing uncertainty surrounding the mean. Distributions were sourced from the literature, along with expert opinion where data on distributions were not available. Assumptions on distributions were made for those remaining parameter estimates, based on the family to which the parameter belonged.

The model took an NHS perspective and a lifetime horizon to capture all differences in lifetime benefits and costs associated with a UCI. The discount rate for future health outcomes and costs was 3.5%, with a 1.5% discount rate tested within a sensitivity analysis. Both these discount rates are recommended by NICE guidelines within their Technology Appraisal Programme [32]. A discount rate of 3% and 5% was also tested within the sensitivity analysis to help compare results between countries, as recommended by The Panel on Cost-effectiveness in Health and Medicine [33]. The model employed a six-month cycle length. All costs were expressed in 2018 UK pound prices.

### Model structure

**Fig. 1:**
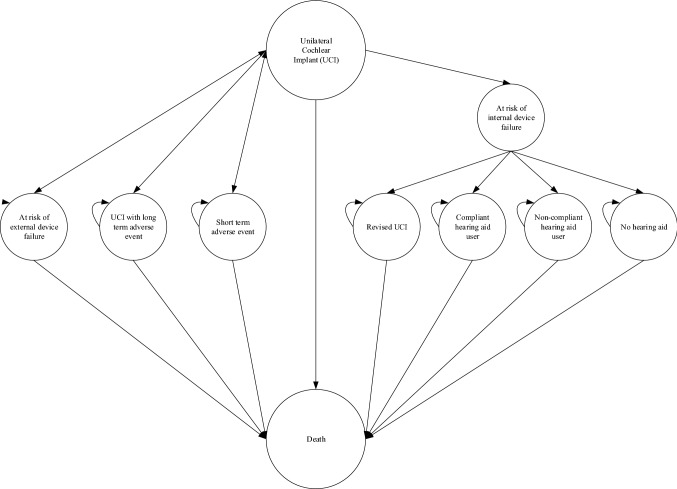
Markov model structure for unilateral cochlear implants

The model explored alternative combinations of interventions and comparators, resulting in several economic evaluation scenarios. These included a UCI versus a hearing aid and a UCI versus no hearing aid. The model incorporated several health states to capture the treatment pathway and potential adverse events and device failures and death from other causes (Fig. 1).

**Fig. 2:**
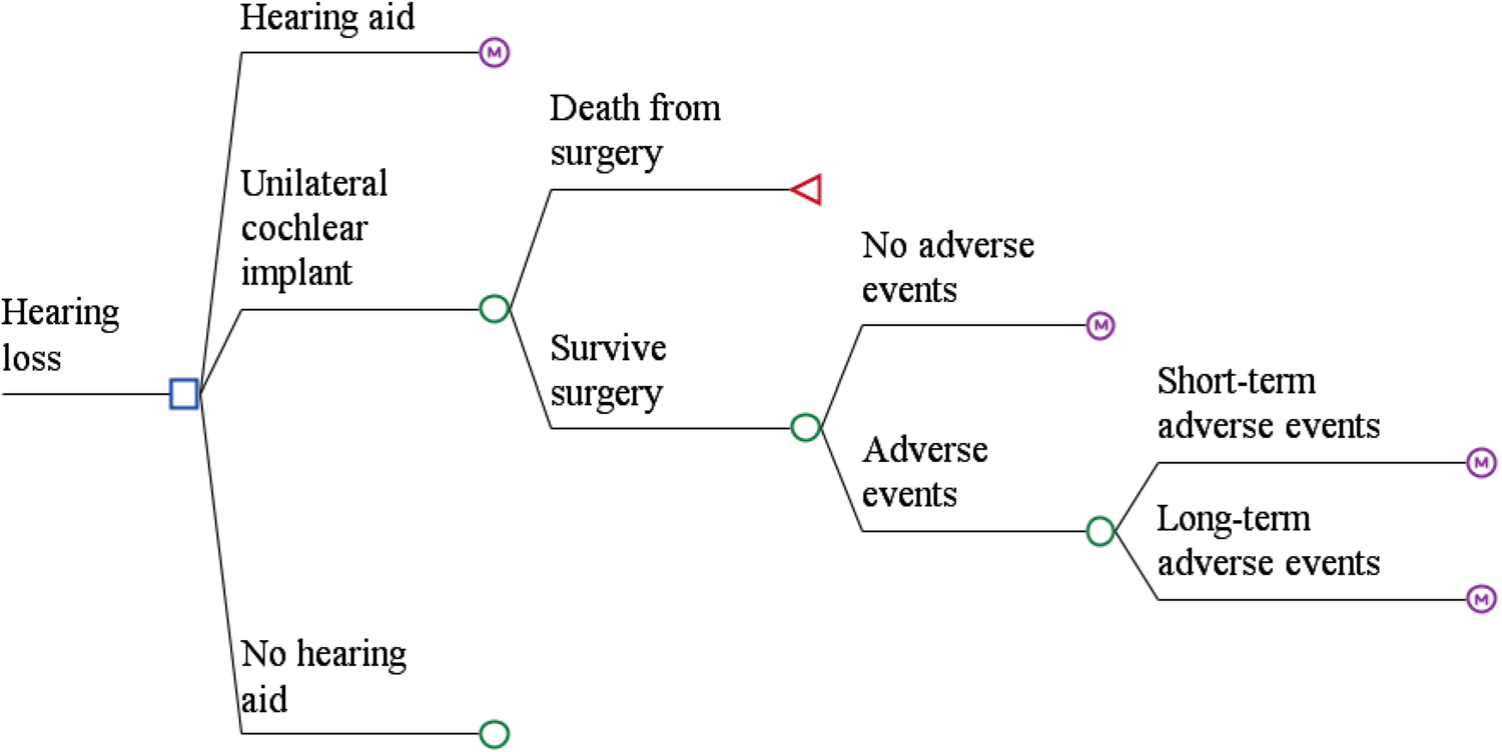
Treatment pathway for adults with severe to profound sensorineural hearing loss

Individuals can remain in their initial state (‘UCI’), or experience an adverse event. These were categorised as either short-term adverse events that lasted one cycle (i.e., six months), or long-term events that lasted for their remaining lifetime (Fig. 2).

Internal and external device failure or external device upgrade can either be immediate (in the first cycle) following surgery, or it can occur over time. This was incorporated into the model using a time-dependent probability calculated from cumulative survival values.

**Fig. 3:**
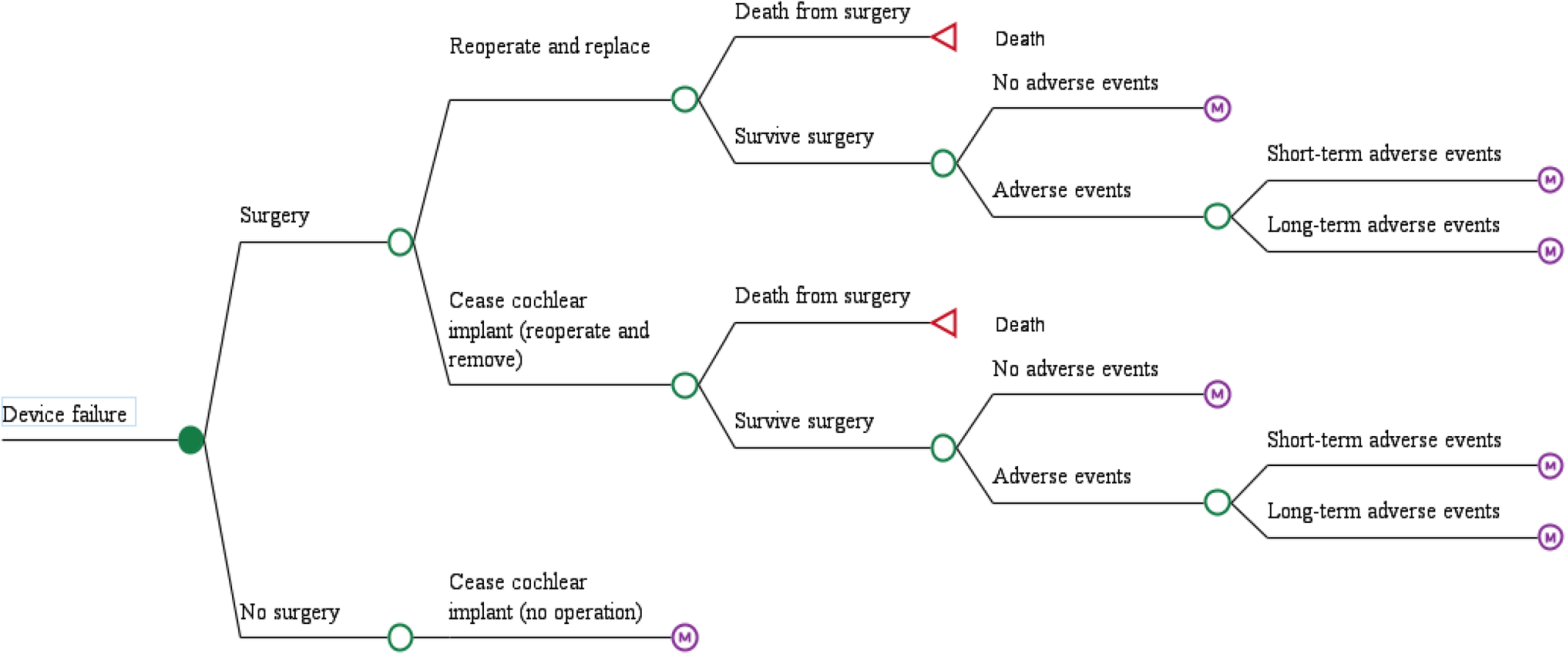
Treatment pathway for patients experiencing and internal device failure

Individuals who experience an internal device failure may choose to re-operate and replace the device (Fig. 3). These individuals may experience an adverse event, which could either be short term or long term. Individuals who experience an internal device failure may also choose to re-operate and remove the device, thereby ceasing to use cochlear implants. Alternatively, they could undertake no operation and remain with a faulty device and cease to use the cochlear implant.

Individuals who experience an external device failure may choose to replace the device or remain with a faulty device and cease to use the cochlear implant. There is no risk of death or adverse events with external device failure. Individuals who experience an external device upgrade will have the device replaced. There is no risk of death or adverse events with external device upgrade.

Patients who cease to use a cochlear implant can either use a hearing aid, where they can be compliant or non-compliant, or they can choose not to use a hearing aid. ‘Death from surgery or natural causes’ is an absorbing health state, accounting for the risk associated with cochlear implant surgery and general all-cause mortality over time in the UK adult population.

### Model assumptions

Several structural assumptions were required within the Markov model. Persons were assumed to have been diagnosed with severe to profound sensorineural hearing loss in both ears. All persons deemed eligible for a cochlear implant were assumed to receive a cochlear implant (i.e., no persons drop off the waiting list before surgery). Any future hearing loss the patient experiences does not impact health outcomes given hearing loss is already severe to profound. Those persons using a hearing aid (instead of a UCI) were either compliant users or non-complaint users.

Several assumptions were made regarding the device itself. Annual equipment maintenance costs start in the second year from receiving a cochlear implant. The internal component of a cochlear implant lasts a lifetime, unless trauma occurs resulting in device failure, and that all cochlear implants are 3 T MRI compatible.^1^ Cochlear implant software upgrade does not provide additional health utility benefits, nor does a cochlear implant impact the prevalence or severity of other health conditions (e.g., depression or dementia). Both these assumptions are likely to be conservative as upgrades could improve hearing and research has linked improved hearing from cochlear implants to improved cognitive function [12]; however, no studies have quantified the impact on utilities. It was also assumed that cochlear implant surgery is never abandoned during the procedure, nor results in death, all initial cochlear implants and re-implants are successful, and that a cochlear implant does not impact life expectancy.

### Model inputs

The Markov model required several underlying parameters related to its structure, device failure, probability of re-implantation, and implant type, among other categories (Table 1). Data were initially identified through a NICE Health Technology Assessment Report on the cost-effectiveness of cochlear implants in the UK; [25] a health technology assessment report produced by Health Quality Ontario on the cost-effectiveness of cochlear implants in Canada; [34] and an unpublished systematic literature review report on the clinical effectiveness of cochlear implants undertaken by Oxford PharmaGenesis Ltd.

**Table 1:** Model parameters

Parameter	Value	Source	Confidence interval	Distribution
Time horizon	Lifetime	Authors assumption based on the expected benefits and costs of a cochlear implant	NA	NA
Annual discount rate	3.5%	NICE guidelines (Technology Appraisal Programme)	1.5%, 5.0%	NA
Average age	52.8 years	Cochlear Limited, based on a distribution of age 18 years and over upon receipt of a cochlear implant in the UK	NA	NA
Proportion of people deemed eligible for a cochlear implant after initial assessment	0.70	Expert opinion	0.372, 0.938	Beta
Probability of a cochlear implant internal failure^a^	0.025	Wang et al. [53]	0.011, 0.040	Beta
Probability of a cochlear implant external failure^a^	0.004	Wang et al. [53]	0.002, 0.018	Beta
Probability a patient elects to discontinue using their cochlear implant	0.077	Kumar et al. [54]	0.009, 0.206	Beta
Proportion of people who receive a benefit from using a hearing aid	0.50	Bond et al. [25] reconfirmed through expert opinion	0.061, 0.939	Beta
Mean lifetime of an acoustic hearing aid	5 years	Bond et al. [25] reconfirmed through expert opinion	1.3 years, 11 years	Gamma
Mean time to sound processor upgrade	106 months	Cochlear Limited Internal Database (2018)	29 months, 232 months	Gamma
Proportion of unilateral candidates adopting a hearing aid and are compliant	0.50	Bond et al. [25] reconfirmed through expert opinion	0.061, 0.939	Beta

^a^Failure rates were derived from a retrospective review of 235 cases of cochlear implant revisions between 1982 and 2011 within the Sydney (Australia) Cochlear Implant Centre [53]. While internal data from Cochlear Limited suggested lower failure rates are associated with Cochlear Limited implants, these may not represent the average failure rate of all manufacturer’s cochlear implants available through the NHS

Literature reviews on transition probabilities and utilities associated with hearing aids and cochlear implants were also conducted up to October 2018 to update and supplement data presented in the three primary sources. Search terms used within the NICE Health Technology Assessment Report were reviewed for their appropriateness, and applied to Ovid Medline and Embase. New search terms were developed for the literature review on utilities. No meta-analyses on utilities were performed due to a high degree of clinical heterogeneity in addition to poor reporting of methods in studies of cochlear implants. Expert opinion was used to fill data gaps associated with resource use and costs.

### Resource use

**Fig. 4:**
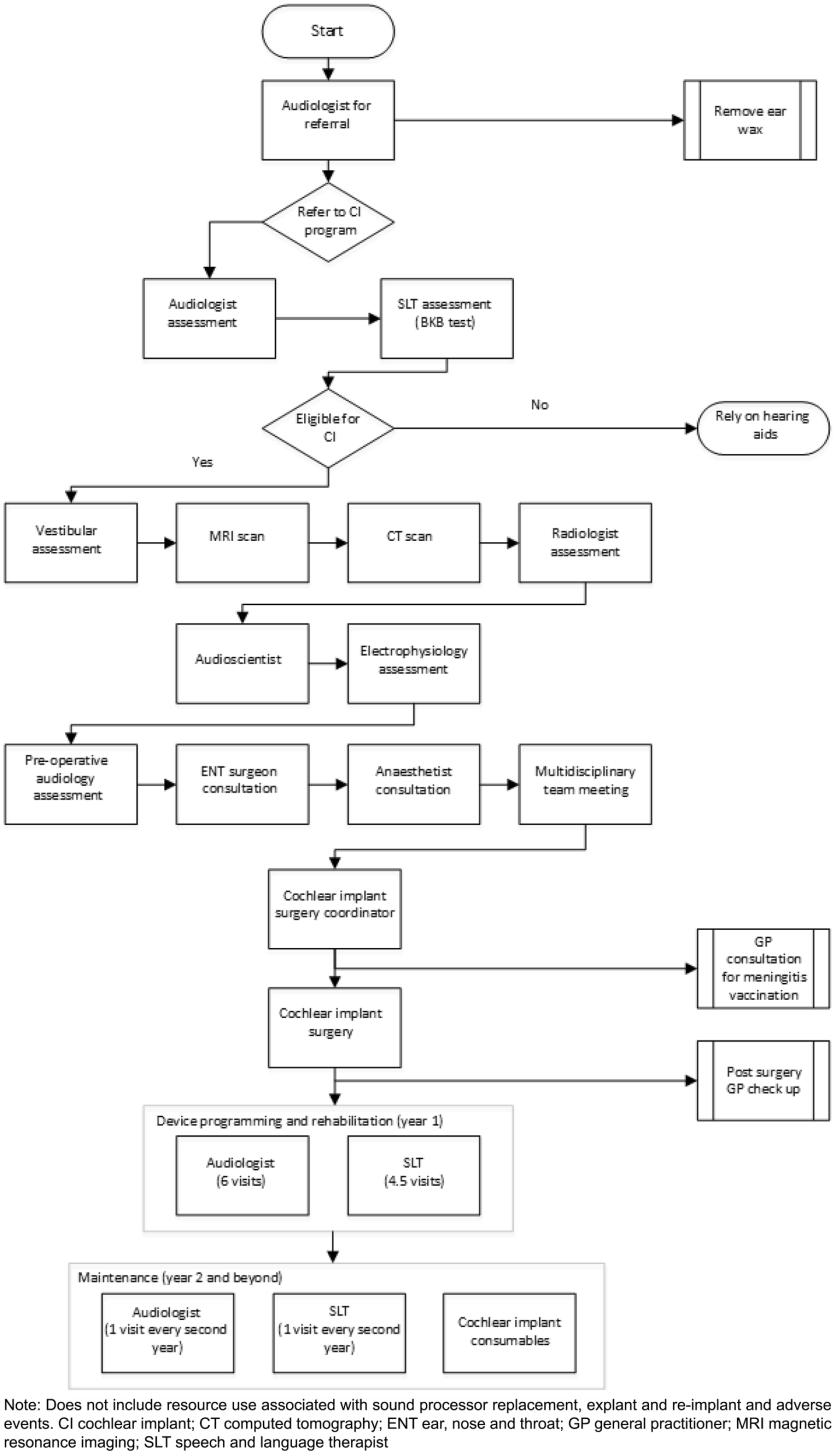
Pathway for resource use associated with a cochlear implant

Resource use was derived from published literature, grey literature and expert opinion. This information was used to develop a treatment pathway that an average patient is expected to take, from receiving an initial referral to a cochlear implant program from an audiologist, to receiving and using a cochlear implant (Fig. 4). Resource use was divided into pre-implant assessment, surgery (the implant device and surgery), device programming and maintenance, sound processor replacement/upgrade, explants and re-implants, and complications (short- and long-term adverse events).

### Utilities

A systematic literature review was undertaken to extract health state utility values relating to hearing loss, cochlear implants and hearing aids in adults aged 18 years and over. While UK studies were preferred, studies from other countries with similar health care systems were also explored. Utilities derived from persons with severe to profound sensorineural hearing loss within a randomised controlled trial were prioritised, along with studies using the HUI3, given this instrument includes hearing and speech domains [35–37].

Utility decrements from severe to profound sensorineural hearing loss were calculated by subtracting the HUI3 utility score of having no cochlear intervention derived from a UK study [5], from the Canadian HUI3 population utility norm. Due to the lack of HUI3 population utility norms for the UK, the model applied the Canadian set of population utility norms (as opposed to either the Australian or US set) given it was considered the most comprehensive and recent. This allowed the model to reduce population norm utilities over time, reflecting poorer quality of life associated with comorbidities and ageing (Table 2).

**Table 2:** Utility values

Health state	Utility	Source
Severe and profound hearing loss prior to a cochlear implant		
Traditional candidates^a^	0.410	UKCISG [5]
Marginal hearing aid users^b^	0.494	
Utility decrement from population utility norms for persons with severe and profound hearing loss		
Traditional candidates	0.439	
Marginal hearing aid users	0.374	Calculated by subtracting the HUI3 utility score of having no cochlear intervention from the Canadian HUI3 population utility norm
Utility increment associated with the intervention received		
Unilateral cochlear implant		
Traditional candidates	0.214	UKCISG [5]
Marginal hearing aid users	0.151	UKCISG [5]

^a^Traditional candidates are those people for whom a hearing aid provided no benefit

^b^Marginal hearing aid users are those persons for whom a hearing aid provided some benefit

A literature review was conducted to identify utility decrements associated with short-term adverse events and long-term adverse events associated with cochlear implant surgery. There were 17 different types of adverse events associated with cochlear implants found within the literature, excluding device complications such as electrode migration. These were allocated to six categories, including infection (skin infections, otitis media), neurological complications (facial palsy, dysgeusia), pain (facial stimulation, other), tinnitus (worsening or new occurrence), vestibular complications (vertigo, dizziness), and other complications (cerebrospinal fluid leak hematoma, atlantoaxial subluxation). Only those with a prevalence of greater than one% were included in the model, which include dysgeusia (taste disturbance), vertigo, tinnitus and wound infection (Table 3). It was assumed that short-term adverse events would last for six months. They were included in the model as a weighted average of disutility using the probability of experiencing an adverse event as weights (Table 4).

**Table 3:** Disutility values

Health state	Disutility	Duration	Source
Short-term adverse events			
Dysgeusia (taste disturbance)	0.020	6 months	Authors assumption
Vertigo	0.033	6 months	Swan et al. [55]
Tinnitus	0.050	6 months	Happich et al. [56]
Wound infection	0.042	6 months	Prosser et al. [57]
Long-term adverse events			
Vertigo	0.033	Lifetime	Swan et al. [55]

**Table 4:** Probability of adverse events

Parameter	Probability	Source
Short-term adverse events		
Dysgeusia (taste disturbances)	0.065	Inverse weighted average of Hanson et al. [58], Jeppesen et al. [59], and Farinetti et al. [60]
Vertigo	0.194	Inverse weighted average of Hanson et al. [58], Jeppesen et al. [59], Farinetti et al. [60], and Venail et al. [61]
Infection	0.015	Inverse weighted average of Hanson et al. [58], Jeppesen et al. [59], Stamatiou et al. [62], Farinetti et al. [60], Venail et al. [61]
Tinnitus	0.036	Inverse weighted average of Jeppesen et al. [59], Farinetti et al. [60] and Venail et al. [61]
Long-term adverse events		
Vertigo	0.014	Inverse weighted average of Hanson et al. [58] and Jeppesen et al. [59]

### Costs

Costs were calculated by multiplying the volume of resource use associated with interventions by their unit cost (Tables 5, 6, 7, 8, 9, 10). Resource use was largely based on clinical expert opinion sought within the development of the clinical pathway. Hearing aid costs depended on the number of hearing aids a patient was using before receiving a UCI. Persons with hearing aids that receive a UCI were assumed to no longer require a hearing aid in the ear receiving the implant and to continue wearing their contralateral hearing aid.

**Table 5:** Pre-implant assessment resource use and unit costs

Resource	No. of visits		Units	Unit type		Unit cost (£)		Source
Referral								
Audiologist	1		1	Consultation		84		NHS National Schedule of Reference Costs [30]
Removing earwax			1	Hours		106		NHS National Schedule of Reference Costs [30]
Stage 1: initial assessment								
Audiologist	1		1.5	Hours		84		NHS National Schedule of Reference Costs [30]
SLT	1		1.5	Hours		96		NHS National Schedule of Reference Costs [30]
Stage 2: testing								
Vestibular assessment and tests			1.5	Hours		86		NHS National Schedule of Reference Costs [30]
Radiologist	1		1	Hours		74		Curtis and Burns [63]
MRI scan			1	Hours		138		NHS National Schedule of Reference Costs [30]
CT scan			1	Hours		88		NHS National Schedule of Reference Costs [30]
Stage 3: electrophysiology								
Audioscientist	1		1	Hours		84		NHS National Schedule of Reference Costs [30]
Electrophysiology assessment			1	Hours		70		Curtis and Burns [63]
Stage 4: medical assessment								
Audiologist pre-operative assessment	1		1.5	Hours		84		NHS National Schedule of Reference Costs [30]
ENT surgeon consultation	1		1	Hours		104		NHS National Schedule of Reference Costs [30]
Anaesthetist consultation	1		1	Hours		130		NHS National Schedule of Reference Costs [30]
Multidisciplinary team meeting - Audiology - SLT - ENT specialist	1		1	Hours		284		Aggregated unit costs using NHS National Schedule of Reference Costs [30]
GP consultation for meningitis vaccination	1		1	Consultation		31		Curtis and Burns [63]
Meningitis vaccination			1	Unit		60		NHS Vaccine Price List
Stage 5: pre-procedural assessment outcome discussion								
Cochlear implant surgery coordinator	1	1			Hours		44	Curtis and Burns [63]

*CT* computerized tomography; *ENT* ear, nose and throat; *GP* general practitioner; *MRI* magnetic resonance imaging; *NHS* national health service; *SLT* speech and language therapist

**Table 6:** Surgery resource use and unit costs

Resource	No. of visits	Units		Unit type	Unit cost (£)	Source
Hospital ward						
Unilateral CI operation—surgical cost	1	1		Visit	5956	Authors calculations using 2018/19 NHS National Tariff: Currencies and Prices [64]^1^
Unilateral CI operation—device cost		1		Implant	16,964	

The 2014–2015 Patient level costing and information systems (PLICS) was used to derive the proportional cost between surgical and device cost, and then applied to the total cost derived from Annex A of the 2018/19 NHS National Tariff: Currencies and Prices

**Table 7:** Hearing aid resource use and unit costs

Resource	No. of visits	Units	Unit type	Unit cost (£)	Source
One hearing aid	1	1	Unit	166	NHS National Schedule of Reference Costs [30]
Pair of hearing aids	1	1	Unit	332	NHS National Schedule of Reference Costs [30]

**Table 8:** Device programming and rehabilitation resource use and unit costs

Resource	No. of visits	Units	Unit type	Unit cost (£)	Source
Initial care—Year 1					
GP medical check	1	1	Consultation	31	Curtis and Burns [63]
CI programming					
Audiologist	6	1.5	Hours	84	NHS National Schedule of Reference Costs [30]
Familiarisation with using the device and aural training					
SLT	4.5	1.5	Hours	96	NHS National Schedule of Reference Costs [30]
Follow-up care—Year 2 and beyond					
Audiologist (tuning visit)	1	1	Hours	84	NHS National Schedule of Reference Costs [30]
SLT (tuning visit)	1	1	Hours	96	NHS National Schedule of Reference Costs [30]
Annual equipment maintenance (batteries, cables, coils and sound processor repairs)		1	Units	328	NHS National Schedule of Reference Costs [30]
CI annual administration	1	1	Hours	44	Curtis and Burns [63]

*CI* cochlear implant; *GP* general practitioner; *SLT* speech and language therapist

**Table 9:** Sound processor replacement / upgrade, explant and re-implant resource use and unit costs

Resource	No. of visits	Units	Unit type	Unit cost (£)	Source
Sound processor replacement/upgrade					
External component	1	1	Unit	5000	Cochlear Limited
Audiologist (tuning visit)	1	1.5	Hours	84	NHS National Schedule of Reference Costs [30]
SLT (tuning visit)	1	1.5	Hours	96	NHS National Schedule of Reference Costs [30]
Explant					
Audiologist (assessment)	1	1.5	Hours	84	NHS National Schedule of Reference Costs [30]
Explant		1	Visit	4253	NHS National Schedule of Reference Costs [30]
Re-implant					
Audiologist pre-operative assessment	1	1.5	Hours	84	NHS National Schedule of Reference Costs [30]
ENT surgeon consultation	1	1	Hours	104	NHS National Schedule of Reference Costs [30]
Anaesthetist consultation	1	1	Hours	130	NHS National Schedule of Reference Costs [30]
Multidisciplinary team meeting - Audiology - SLT - ENT	1	1	Hours	284	Aggregated unit costs using NHS National Schedule of Reference Costs [30]
Cochlear implant surgery coordinator	1	1	Hours	44	Curtis and Burns [63]
Unilateral CI operation—surgical cost	1	1	Visit	5956	Authors calculations using NHS National Schedule of Reference Costs [30]^a^
GP medical check	1	1	Consultation	31	Curtis and Burns [63]

*CI* cochlear implant; *ENT* ear, nose and throat; *GP* general practitioner; *SLT* speech and language therapist

^a^The 2014–2015 Patient level costing and information systems (PLICS) was used to derive the proportional cost between surgical and device cost, and then applied to the total cost derived from Annex A of the National tariff payment system 2017–2018 and 2018–2019

**Table 10:** Resource use and unit costs associated with short-term and long-term adverse events

Parameter	No. of visits	Units	Unit type	Unit cost (£)	Source
*Short-term adverse events*					
Taste disturbances, Vertigo, Tinnitus					
GP visit	1	1	Consultation	31	Curtis and Burns [63]
Infection					
GP visit	1	1	Consultation	31	Curtis and Burns [63]
Antibiotics		1	Course	10	Based on NHS prescription charge [65] and Amoxicillin (Cap 500 mg) [66]
*Long-term adverse events*					
Vertigo					
GP visit	1	1	Consultation	31	NHS National Schedule of Reference Costs [30]

Short-term adverse events are defined as those lasting six months or less. Long-term adverse events are defined as those lasting a lifetime

Unit costs were derived from clinical expert opinion, literature reviews, NHS National Schedule of Reference Costs, NHS National Tariff: Currencies and Prices, the Personal Social Services Research Unit, and literature.

### Sensitivity analyses

A sensitivity analysis of key model parameters was undertaken to assess the sensitivity of the ICERs to key model parameters. Parameters were chosen based on their expected level of uncertainty and potential influence on ICERs. Their range was dictated by the 95% confidence interval around the estimate (where available), while an arbitrary range was determined for all other parameters [38].

A probabilistic analysis using Monte Carlo simulation was also conducted, using distributions attached to each model parameter, with 10,000 samples drawn at random from these distributions to calculate a distribution of ICERs. Fixed random number seeds were used to minimise random simulation error [39]. A cost-effectiveness acceptability curve (CEAC) was produced to determine the probability of the ICERs being cost-effective across the assumed cost-effectiveness threshold of £20,000 per QALY [38].

### Model validation

Model validation was undertaken using the Assessment of the Validation Status of Health-Economic decision models (AdViISHE) tool [40]. Internal consistency was checked by ensuring probabilities lied between zero and one and probabilities following a chance node summed to one. Two additional people reviewed the mathematical logic of the model, including equations, coding and model inputs. We used null and extreme values within a sensitivity analysis to determine whether subsequent results meet apriori expectations.

The model was checked for external consistency by first comparing ICERs using inputs derived from the NICE Health Technology Assessment Report. This allowed the structure and mathematical logic of the model to be tested, while holding differences in model inputs constant. ICERs generated using model inputs derived within this study were also compared. The model was also validated by clinical experts on whether the assumptions and structure of the model were reliable and could be reasonably explained.

## Results

### Base case results

A UCI for someone that had previously worn a hearing aid in the ear that received the implant led to an average incremental lifetime cost increase of £37,988, and an additional 3.18 QALYs. This equates to an ICER of £11,946 per QALY gained.

A UCI for someone that had not previously worn a hearing aid in the ear that received the implant led to an average incremental lifetime cost increase of £38,449 and an additional 3.66 QALYs. This equates to an ICER of £10,499 per QALY gained (see Table 11). The improved ICER comes from the greater benefit from a UCI, given no benefit from a hearing aid had been received prior.

**Table 11:** Cost-effectiveness results

Strategy	Reference case		
	Cost (£)	QALY	ICER (£ per QALY)
*Deterministic results*			
Hearing aids^a^			
Hearing aid	464	6.78	
Unilateral cochlear implant	38,449	9.96	
Difference	37,985	3.18	11,946
No hearing aids^b^			
No hearing aid	0	6.26	
Unilateral cochlear implant	38,449	9.92	
Difference	38,449	3.66	10,499
*Probabilistic results*^c^			
Hearing aids			
Mean difference	38,465	3.18	12,390
Confidence interval (2.5%)	20,547	3.46	5,943
Confidence interval (97.5%)	62,233	2.68	23,261
No hearing aids			
Mean difference	38,807	3.66	10,634
Confidence interval (2.5%)	20,307	3.63	5,594
Confidence interval (97.5%)	69,113	3.64	19,013

*ICER* incremental cost-effectiveness ratio; *QALY* quality adjusted life years

^a^Patients that wore a hearing aid prior to a cochlear implant and received some benefit (score of 1% to 50% on a BKB test)

^b^Patients that did not wear a hearing aid prior to a cochlear implant because they did not receive a benefit (score of zero on a BKB test)

^c^Derived from Monte Carlo simulation using 10,000 iterations

### Sensitivity analysis

**Fig. 5:**
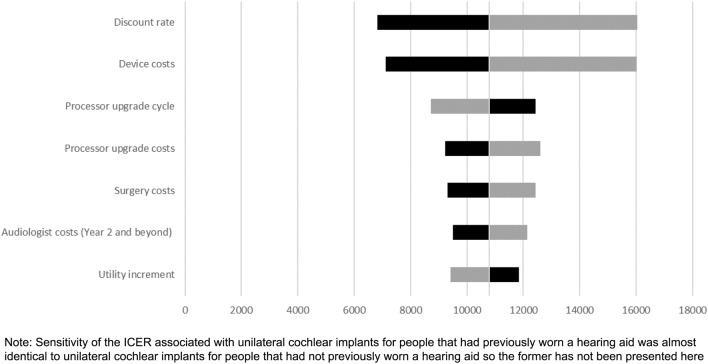
Sensitivity of the ICER for a unilateral cochlear implant compared to no hearing aids

A probabilistic sensitivity analysis showed the ICER associated with a UCI compared to a hearing aid or no hearing aid was sensitive to the discount rate, surgery and device costs, processor upgrade cost and processor cycle time, audiologist costs after the implant, and the utility increment associated with a UCI (Fig. 5).

**Fig. 6:**
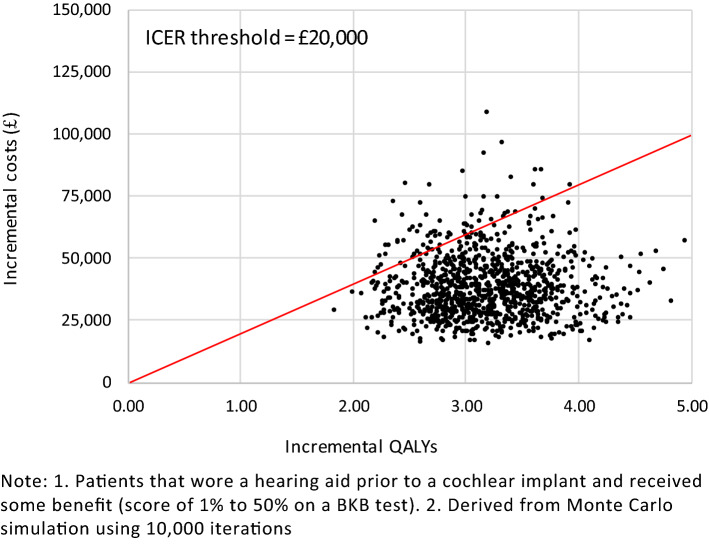
Mean differences in costs and QALYs for a unilateral cochlear implant vs a hearing aid

**Fig. 7:**
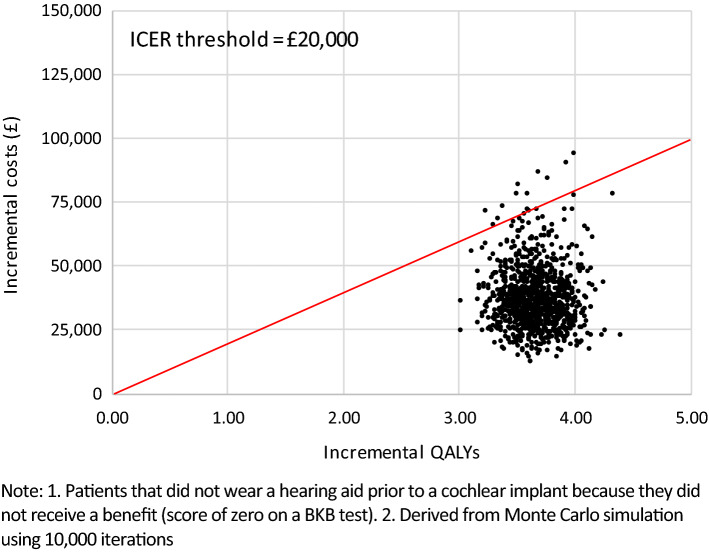
Mean differences in costs and QALYs for a unilateral cochlear implant vs no hearing aid

**Fig. 8:**
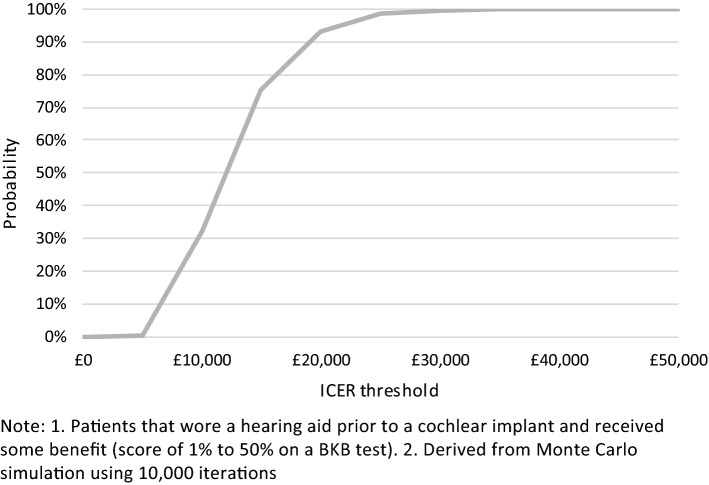
Cost-effectiveness acceptability curve for a unilateral cochlear implant vs a hearing aid

**Fig. 9:**
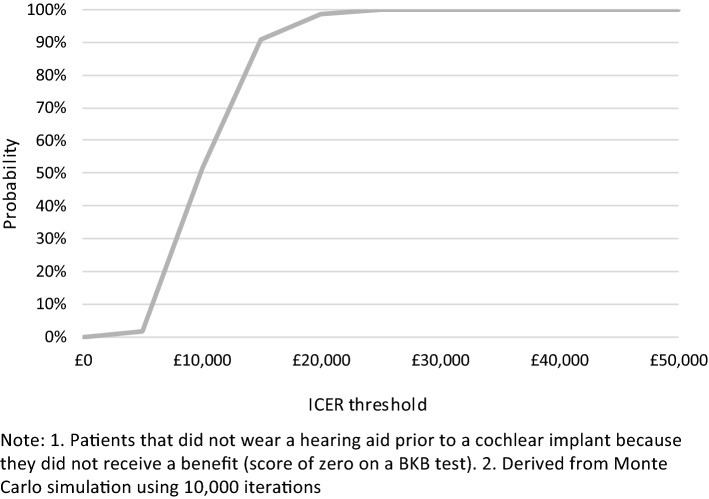
Cost-effectiveness acceptability curve for a unilateral cochlear implant vs no hearing aid

All simulated results for a UCI in the probabilistic sensitivity analysis (PSA) fell into the northeast quadrant of the cost-effectiveness plane, indicating that a UCI is more expensive but also more effective (Figs. 6, 7). Most simulated mean differences in costs and QALYs are below the £20,000 per QALY gained threshold, indicating a UCI compared to a hearing aid, or no hearing aid, is likely to be cost-effective. CEACs show that a UCI has an 93.0% likelihood of being cost-effective when compared to a hearing aid, and a 98.7% likelihood of being cost-effective when compared to no hearing aid (Figs. 8, 9).

## Discussion

Modelling results suggest a UCI for adults with severe to profound hearing loss is cost-effective when compared to a hearing aid or no hearing aid. ICERs were well below the NICE cost-effectiveness threshold and CEACs suggest the probability of UCIs being cost-effective is between 93.0% and 98.7%. These results align with the NICE Health Technology Assessment Report conducted in 2009 [25]. It found the ICER for a UCI compared to no UCI was £14,163 per QALY for adults aged 50 years, with a 100% probability of being cost-effective at a £20,000 per QALY threshold.

Differences in the ICERs reported between the two studies may relate to changes in clinical pathways, resource use and unit costs. Our incremental lifetime costs were up to £4493 more (for someone previously not wearing a hearing aid) compared to the estimate in the NICE Health Technology Assessment Report. While pre-surgical costs and post-surgical costs were lower within our study, there were greater costs associated with cochlear implant surgery, device costs and hearing aids. The largest difference was in surgery and device costs, with our study estimating an additional £3142 and £2352, respectively.

Our study developed a likely pre-surgical and post-procedural pathway currently operating in the UK, and applied unit costs derived from the NHS to expected resource use, including surgery and device costs. The UK Health Technology Report study estimated resource use and costs from a UK Cochlear Implant Study Group report conducted in 2004 [41]. That study relied on estimated cost information sourced from five NHS hospitals up to eight years before 1999, along with clinical and hospital accountant consultations.

Differences in methods and assumptions have also impacted results. This study estimated a patient is around three years older when they receive a cochlear implant compared to the NICE Health Technology Assessment Report, and there were differences in the number and types of adverse events a patient could experience. This study undertook a comprehensive literature review on adverse events to populate the model, while the NICE Health Technology Assessment Report relied on two studies to populate their model. The NICE Health Technology Assessment Report assumed reduced costs for internal device failure, repairs and replacement within a warranty period, which this study did not.

Differing Markov model structures may have also impacted results. The NICE Health Technology Assessment Report allowed voluntary non-use of the cochlear implant, allowing UCI recipients to revert to their original hearing support. Our model allowed UCI recipients to revert to either their hearing aid or no hearing aid, but only if the device failed internally. Furthermore, our model produced two separate ICERs, one for adults with some benefit from hearing aids and another for adults with no benefit from hearing aids prior to receiving a UCI. The NICE Health Technology Assessment Report reported one ICER for all adults.

This study found ICERs were sensitive to the proportion of people eligible for a UCI. This has implications for improving the awareness of access to UCIs after the change in NICE criteria. An influx of people seeking a UCI may increase the ICER if they are deemed ineligible through initial testing. Increased use of audiologists and speech and language therapists would increase costs without a commensurate increase in health outcomes. Improving the quality of referrals from referring clinics through better data collection and education, and carefully managing expectations regarding access to UCIs, is therefore warranted.

### Limitations

While a UCI was estimated to be cost-effective, QALYs may have been underestimated given the most reliable utility estimates were taken from a study completed two decades ago [5]. The introduction of digital processors alongside the use of dual microphones in 2004 represented a major leap forward in cochlear implant technology, helping recipients with directional hearing. Cochlear implant recipients now experience improved hearing in background noise due to the introduction of technological improvements such as input (pre-)processing of the sound signal [42, 43]. The NICE has recommended future research focus on measuring the benefits of bilateral cochlear implants compared to UCIs for adults and children.

Our model represents a simplification of the complex interaction between hearing loss and quality of life. Cost savings were not included with reduced familial support, such as avoiding the need to learn sign language or the potential increase in QALYs for others from the avoidance of stress associated with hearing loss of a family member.

Despite research showing severe to profound hearing loss reduces productivity [44–49], no studies were found that measured the potential increase in productivity from a cochlear implant. One study included potential changes in productivity within their economic evaluation, although this related only to the expected productivity loss associated with having to take time off work for audiology assessments and surgery [34].

The impact of hearing loss on the severity and onset of dementia were also not considered. While several studies have evaluated the impact of hearing aids on cognitive function [50], no studies have evaluated the impacts of improved hearing from cochlear implants on dementia. One study conducted in France suggests cochlear implants may improve cognitive function [12]. It recruited 94 patients aged between 65 and85 years old and provided them with a UCI and two sessions with a speech therapist each week for six months or more. Around 80% of the patients with the poorest Mini-Mental State Examination scores before implantation improved their cognitive function one year after implantation. However, the study could not separate the effects of speech perception improvement from the cochlear implant and the cognitive training that was also provided to patients [66].

If empirically validated, a reduction in dementia risk from cochlear implants would represent a significant benefit. One study has estimated that even delaying the onset of dementia by one year would decrease the prevalence of dementia around the world by 10% in 2050 [51]. Dementia currently costs over 1 trillion U.S. dollars per year and is expected to grow as the population ages [52].

## Conclusion

This study demonstrates that UCIs remain cost-effective since the UK Health Technology Report associated with the original NICE guidance was completed in 2009, despite changes to clinical practice and increased healthcare unit costs. Investment in improved awareness among clinicians and those with severe to profound hearing loss on access to, and potential benefits from, UCIs would further enhance access and improve quality of life and overall social welfare.

## Footnotes

1 Many cochlear implants currently in use are not 3 T compatible. We made this assumption because nearly all new cochlear implants will be 3 T compatible soon.
